# Probabilistic Aspects of Modeling and Analysis of Grinding Wheel Wear

**DOI:** 10.3390/ma15175920

**Published:** 2022-08-26

**Authors:** Wojciech Kacalak, Dariusz Lipiński, Filip Szafraniec, Kamil Banaszek, Łukasz Rypina

**Affiliations:** 1 Faculty of Mechanical Engineering, Koszalin University of Technology, 75-620 Koszalin, Poland; 2 Doctoral School, Koszalin University of Technology, 75-453 Koszalin, Poland

**Keywords:** grinding, grinding wheel, wear, attritious wear, fracturing, volumetric wear, modeling, analysis, statistics, probability

## Abstract

In this article, the methodology of using probabilistic models of the grinding tool wear process is presented. Probabilistic modeling with empirical data allowed determining the values of other important process features. Among them, the distribution of active grains lifetime or distribution of cumulative attritious wear of the grinding grain apex could be distinguished. The results of modeling and wear analysis of grinding wheels as well as experimental results on peripheral grinding with zoned grinding wheels are presented. The analyzed grinding wheels consisted of three layers: two identical external layers with conventional structure and one internal layer containing the addition of abrasive aggregates. The external layers were profiled by chamfering the edges. As a result, their nominal surfaces were conical. The internal layer had a cylindrical shape and was designed for smoothing the surface after machining with external part. The tools were designed to increase the grinding efficiency and hence a good quality of machined surfaces could be acquired. For the experimental tests, the Ti6Al4V titanium alloy was used. It was found that the change in the shape and position of the grinding zone, as a result of volumetric wheel wear, caused a significant change in fracturing intensity. In the case of multilayer grinding tools, the wear process depends on the physical properties of each layer and their participation during machining of the workpiece. The presented methodology could be applied to a study on the machining process stages, which concerns temporary states and their variability according to the machining time.This makes it possible to reduce the cost of developing new tools dedicated to specific applications.

## 1. Introduction

Surface grinding is usually the last step in the manufacturing process, and is most often used as a fine machining step with small material allowances. Its purpose is to create the required geometrical parameters for metals and their alloys, ceramics, or composites [1]. The properties of many alloys and composites, favorable for their applications, often have a negative impact on their machining. Material properties that hinder machining processes include [2,3,4]: (i) high mechanical strength at increased temperatures, which causes elastic and plastic deformations in the grinding zone rather than shearing, (ii) the ability to work hardening and recrystallization at elevated temperatures which causes an increase in mechanical interactions and vibrations (chatter) during machining, (iii) high reactivity with abrasive grain material, which leads to their adhesion on the cutting edge of the tool, and thus to increased wear, (iv) low thermal conductivity, resulting in concentrated energy in a smaller volume, intensifying thermal damage to the surface layer of workpieces or accelerating tool wear, (v) high value of the friction coefficient, which depends on the type of materials in the contact zone, leading to an increase in the specific energy value of the processes.

The above-mentioned properties of the materials lead to the intensification of the wear processes of abrasive tools. They could be analyzed at the micro- and macroscale. The microscale phenomena have been determined for single grains. Among them, the grain attrition, binder fracture or adhesion of the processed material could be distinguished. Attritious wear of the abrasive grain leads to an increase in its contact area with the workpiece. As a result, an increase of the value of grinding force components were observed [5]. Furthermore, that leads to the fractures of whole abrasive grains from the active surfaces of the tools and the cracks occur mainly in the bond bridges [6]. That process continues and the flat areas could be observed on that grinding wheel active surface [7,8]. The phenomena is cumulative and in total leads to the macro wear of the grinding wheel.

The interchanging processes of wear and self-sharpening of abrasive tools are associated with the crushing of grains or their fragments. That changes the radius, and thus the tool is unbalanced, which causes vibrations during the grinding process [9]. They lead to further volumetric changes of the grinding wheels and could be observed on ground surfaces as chatter effect. Moreover, these vibrations result in a local increase of the grinding force and thermal damages of the machined surfaces [10]. In general, the wear of abrasive grains by blunting their sharp tips or fracturing leads to a reduction in the machining potential of the whole grinding tool, as was shown during the analysis of the Shos parameter value [11,12].

Modeling of the grinding process in terms of the durability of abrasive tools is a difficult task. Analytical models have a limitation because of the unknown distribution and orientation of cutting grains on the active surface of the grinding tool. The reconstruction of a part of the tool and not the whole wheel, as an input geometry into the simulations, could be a time saving solution. The process could be performed using optical methods [13]. However, that may not provide sufficient information about the topography of the rest of the tool. Hence the probabilistic models were created. The total tool wear could be expressed as the product of the fracture probability, the average weight of the abrasive grain, and the total number of contacts between the grains and the ground surface [6]. The work [14] presents probabilistic models of abrasive tools in which the position of active vertices was expressed by the known probability density function. However, the tool wear phenomenon was considered only in the case of geometric changes of the grain peaks. Furthermore, in the case of probabilistic modeling and simulations of the grinding tool wear processes, in the work [15], the authors proposed probability distributions and their formulas for the grit protrusion height. It was shown that active grains heights have normal distribution for dressed, “as received” wheel, and binomial probability distribution for the worn, “end of life” tool after processing of Inconel 718 alloy. In the work [16], the authors presented a formula for calculation of the active grains number which was a result of probability that the abrasive grain will be in the active band of the tool topography cross section area. According to the authors, the probability of grain fracture was estimated, as a common distributions range of grains contact and their reduced strength. In [17], the authors calculated the radial tool wear using the probabilistic model of contact between grain active peaks and the workpiece.

Among the numerical methods for simulations of physical phenomena, modeling of machining processes occupies an important place. The calculations could be carried out in order to predict the output values of the grinding process [18,19]: (i) values characterizing the load of the tool and the workpiece (the value of the grinding force or the specific energy of the process), (ii) parameters of the machining layer condition (residual stress, roughness, microhardness, surface roughness), (iii) condition of the active surface of the tool (grinding coefficient, volumetric wear, change of machining potential), (iv) chips shapes. The numerical methods applied for the grinding process include [20]: the finite element method (FEM), the smoothed-particle hydrodynamics (SPH) method, the analysis of regression models and artificial neural networks [21]. They could be applied for the simulation of the entire grinding wheel or a single abrasive grain. Calculations performed for grinding with a single abrasive grain could provide information about the phenomena in the material separation zone. For example, to learn about the differences of the chip formation and side material flow for conventional and aggregate grains the FEM method was used [22]. That approach was also used in order to understand the mechanism of the grit cracking, which was presented in the example of the cBN grains [23].

Increasing the credibility of numerical calculations for the grinding process is related to the precision of the mathematical models for phenomena such as an increase of temperature or forces. However, also the details of modeled grinding wheel active surface or the single grain are important [24,25]. The methods of recreating the topography of the tools required the grain models based on regular geometric solids. Then they were arranged in the active topography with some references to the measured tools [13,20]. The presented solutions increased the compatibility of the calculated and experimental results because the initial conditions were closer to the real ones [24]. However, as the grinding process progresses,  wear and micro-sharpening processes occur on the active surface of the tools, which directly affect their machining potential and thus the ground surface quality [11]. This means that the shape and proportion of active abrasive grains would vary. It is important to approximate the occurrence of active and potentially active grains. That depends on the grinding depth deviations caused by [1]: (i) the trajectory of the grains which is a result of process kinematics, (ii) initial surface roughness of the machined parts, (iii) vibrations of the tool, and (iv) nonuniform composition and properties of ground material.

The final shape of the grinding wheel active surface is the effect of many random processes [26]. During the production of the grains, crushing large crystals makes their shape irregular. Moreover, the grinding wheels manufacturing process consists of mixing, pressing and sintering powders in a mold, which causes random distribution and orientation of particles in the volume of the tool [27]. Additionally the differences in the specific gravity of the grains and the bond determine the grain distribution. Despite successful attempts to create a deterministic abrasive texture on the active surface of the tool [28], conventional methods are most often used for industrial applications. Moreover, the stochastic nature of the grinding tool active topography could be also a result of the dressing process, where the grains crush into the irregular shapes during the contact with the dresser.

When starting the simulation, it is possible to use a close-to-real, reconstructed topography of the grinding tool active surface. Additionally, it could be assumed that the specific energy in abrasive and erosive machining processes is usually in a range from 10 to 1000 J/mm^3^ with the temperature exceeding 1200 °C in micro volumes [19]. The result of the high energy consumption of manufacturing processes, leading to the high values of forces, are the deformations of the object, tool, and machining system. The thermal load of the grit leads to a reduction in the fracture toughness of abrasives [19]. The propagation of the grains fractures depends on the particle size which is random [29]. The above-described relationships create the need to take into account the lifetime of the abrasive grains, which is varying due to the physical phenomena. That could be used as an additional factor in the already existing simulation methods or for estimating the life of an abrasive tool. In order to do that the probabilistic models could be used.

The review of the methods of analysis of the grinding wheels volumetric wear used so far indicates the need to take into account the probabilistic features of the process influencing the changes in the intensity of abrasive grain fractures in the wear models of abrasive tools. The analysis of the effects of these processes is crucial for grinding control and is a problem of great technological and economic importance. The research presents probabilistic models of grinding wheel wear processes, taking into account: the working time distribution of active grains, the intensity of abrasive grain fracturing processes as well as the total wear surface and volumetric wear of abrasive tools. The models of the grinding wheel wear processes include the phenomena of grain (bond) fracture and attritious wear of the abrasive grains. As a result of experimental and numerical tests, it was shown that the change in the shape and position of the grinding zone, resulting from the volumetric wear, caused a significant change in the intensity of abrasive grain fracturing. In the analyzed case of zone-diversified tools, this process depended on the properties of individual zone and their variable activity in the shaping of the ground surface. The developed methodology of the grinding wheel condition analysis allowed describing their temporary states and changes with the operation time. Their application would allow reducing the costs of developing new tools dedicated to specific processes.

## 2. Probabilistic Models of Grinding Wheel Wear Processes

### 2.1. Modeling Assumptions

The mathematical models of probabilistic wear processes were built on the assumption that the wear of abrasive tools depends mainly on the attrition and fracture strength of the grains. The grinding process and the tool wear depend on the changing state of the grinding wheel active surface and they are not stationary. Developing models, the following assumptions were drawn:

- the grains pull-out and fracturing of their vertexes occur simultaneously,
- the attritious wear of the grit leads to the increased area of the contact, thus increased grinding force,
- the process of grain fracture, as a result of exceeding the mechanical strength, depends on the distribution of the limit grain strength and the distribution of the grain load which is variable during subsequent contacts of the grain with the workpiece,
- in working conditions with intensive self-sharpening, the probability of grain fracture (pull-out) does not depend on the time of its operation,
- in the operating conditions of a grinding wheel with limited self-sharpening effect, the average time to grains fracture, when their operating time is known, may depend on the total operating time of the abrasive grain,
- constant intensity of fractures (pull-outs) could be described by the exponential distribution of grain durability,
- the decreasing intensity of fractures (pull-outs) could be described by the log-normal distribution of grain durability,
- the increasing intensity of fractures (pull-outs) could be described by the right-tail normal distribution values, Weibull or gamma distribution.

It was also taken into account that the thermal and mechanical load of the abrasive grains, their mechanical strength and resistance to the accumulation of loads, as well as the force necessary to fracture the grain, were randomly changing values.

### 2.2. Modeling of Abrasive Grains Fracture (Pull-Outs)

In the models of grain fracturing processes, it was assumed that the grain working time from 0 to $t_{z}$ is the grain durability, and R(t)=P(t<tz) is the grain durability distribution. The residual cumulative distribution function of the persistence Rg(t), i.e., the time left at this moment until the grain is fractured $t_{az}=t_{z}-t_{a}$ is as follows:

(1) $$R_{g}\left(t\right)=P\left(t_{az}<t|t_{z}>t_{a}\right)=\frac{P(t_{az}<t\cup t_{z}{>}_{a})}{P(t_{z}>t_{a})}$$

which gives:

(2) $$R_{g}\left(t\right)=\frac{R\left(t+t_{a}\right)-R\left(t_{a}\right)}{a-R(t_{a})}$$

The probability density $r_{g}\left(t\right)$ is following:

(3) $$r_{g}\left(t\right)=\frac{r(t+t_{a})}{1-R(t_{a})}$$

The mean value of the residual distribution, i.e., the time left at the moment to fracture the grains, is as follows:

(4) $${\bar{t}}_{az}=\frac{1}{1-R(t_{a})}\int_{0}^{\infty }\left(1-R\left(t\right)\right)dt$$

The intensity of fracturing is related to the function $R_{g}\left(t\right)$. It was described by the formula:

(5) $$\lambda \left(t_{a}\right)=\frac{r(t_{a})}{1-R(t_{a})}$$

For grains that worked $t_{a}$ time ($t_{a}$ grain age), the probability that they would be fractured in the time interval $\left(t_{a},t_{a}+\Delta t_{a}\right)$ is as follows:

(6) $$P\left(t_{az}<t_{z}<t_{a}+\Delta t\right)=\frac{R\left(t_{a}+\Delta t\right)-R\left(t_{a}\right)}{1-R(t_{a})}$$

This probability related to the length of the time interval Δt is equal to:

(7) $$P_{\Delta t}=\frac{P(t_{az}<t_{z}<t_{a}+\Delta t)}{\Delta t}=\frac{1}{1-R(t_{a})}\frac{R\left(t_{a}+\Delta t\right)-R\left(t_{a}\right)}{1-R(t_{a})}$$

and for Δt→0 it goes to $\lambda (t_{a})$.

Knowing the intensity of fracturing, it is possible to determine the distribution of the grains durability:

(8) $$R\left(t\right)=1-e^{\int_{0}^{t}\lambda \left(t_{a}\right)dt}$$

Experimental determination of the intensity of fracturing is usually easier than the determination of the grain durability distribution.

#### 2.2.1. Constant Intensity of Fracturing

A constant intensity of fracture is characteristic of a stable machining process in plane or cylindrical grinding operations, except for contour grinding. In that case, the exponential distribution could be used to describe the fracture process. Then:

(9) $$R\left(t\right)=1-e^{-\lambda t},\;\lambda >0$$

(10) $$r\left(t\right)=\lambda e^{-\lambda t}$$

(11) $$\lambda (t_{a})=\lambda =const$$

In this case, the residual life time of the grain $t_{az}$ has the same distribution as the time tz. This means that the probability of grain fracture does not depend on the time the grain has been active. In the grinding processes, this corresponds to a situation in which there is a significant variation in the load of the grains and a significant variability of their strength and fixing forces in the binder. The more open the structure of the grinding wheel and the greater fracturing of the grain vertexes, resulting for example from rough dressing of the grinding wheel, the more the fracturing intensity could be considered to be constant.

#### 2.2.2. Decreasing Fracturing Intensity

The decreasing intensity of grain fracture, especially for high grain durability, could be described by the log-normal distribution. Then:

(12) $$r\left(t\right)=\frac{1}{t\sigma \sqrt{2\pi }}e^{\frac{-1}{2\sigma^{2}}{\left(logt-\mu \right)}^{2}}$$

where: σ>0, −∞<μ<∞.

#### 2.2.3. Increasing Fracturing Intensity

Normal one tailed distribution could be used for modeling the increasing intensity of fracturing, which usually corresponds to the work of cut-off grinding wheels, when with a decreasing diameter, the grinding wheel and its circumference increases the load on the grains. In this case, the density function of the fracturing probability takes the form:

(13) $$r\left(t\right)=\frac{1}{a\sigma \sqrt{2\pi }}e^{\frac{-{\left(t-\mu \right)}^{2}}{2\sigma^{2}}},\;\sigma >0,\;\mu >0,$$

where: $a=\frac{1}{\sqrt{2\pi }}\int_{-\frac{\mu }{\sigma }}^{\infty }e^{\frac{-u^{2}}{2}}du$ is a normalizing factor, taking into account that, że t>0.

### 2.3. Probabilistic Models of Abrasive Grains Load

The grain load was taken as the sum of the following components:

- the average value of the abrasive grain load:
  (14) $${\bar{F}}_{z}=E\left[F_{z}\left(t\right)\right]$$
- random component $F_{p}\left(t\right)$ with the expected value equal to zero and the variance e $D^{2}\left[F_{p},t\right]$ variable with the machining time:
  (15) $$F_{z}\left(t\right)=E\left[F_{z}\left(t\right)\right]+F_{p}\left(t\right)$$

To determine the probability distribution of grain fractures as a result of the grains loads exceeding the limit level of grain strength $w_{z}$, by carrying out the $F_{z}\left(t\right)$ process in a finite period of time, it is necessary to determine the distribution of the exceeding numbers of the limit level. When it could be stated that the level of $w_{z}$ is high, and the average number of exceedances in the time interval is small, then the successive exceedances could be considered to be independent. This case also occurs when the variability of the load is significant. That could be described by the stationary Poisson process, in which the probability that in the time interval $t_{a}=t_{a}-\Delta t$ there would be not a single excess of $w_{z}$ value:

(16) $$P\left(n_{p}\left(t_{a},w_{z}\right)\right)=e^{-t_{a}n_{p}}$$

where: $n_{p}$—expected number of exceeding the level $w_{z}$ in a unit of time.

In the case when the process $F_{p}\left(t\right)$ has a normal distribution, then the distribution of the average number exceeding the load level $w_{z}$, by the process $F_{z}\left(t\right)$, could be represented as follows:

(17) $$n_{p}=E\left[{\bar{F}}_{z}\right]e^{\frac{{\left({\bar{w}}_{z}-{\bar{p}}_{z}\right)}^{2}}{2D^{2}\left[F_{p}\right]}}$$

where $E[{\bar{F}}_{z}]$ denotes the expected value of exceeding the ${\bar{F}}_{z}$ level in a time unit by the process $F_{z}\left(t\right)$, and $D^{2}\left(F_{p}\right)$ denotes the variances of the process $F_{p}\left(t\right)$.

The above relations describe grain fracturing as a result of exceeding the strength of the grains themselves or the bond bridges toughness. Grain may also fracture or crack as a result of a lower load if the strength of its joints in the binder has decreased. That could be a result of the destructive effect of earlier loads. The high brittleness of the abrasive grains and bond bridges is the reason why the strength wear occurs more often as a result of exceeding the immediate strength than the fatigue strength.

In the case of polycrystalline grains or micro-aggregates, the fracturing processes could become more complicated as the micro-fracturing becomes a typical wear feature. Then, in the developed models, smaller intensities of chipping were introduced (based on the research of the processes), and the intensity of abrasive wear was reduced. However, it should be taken into account that the number of grain vertexes increases and the average cross-section (thickness) of the cut layer decreases. That increases the specific grinding energy but to a much smaller extent than for attritious wear of the grains.

### 2.4. Distribution of Active Grains Cutting Time

The grain fracture processes changes the number of active grains and their activity time. The variable features are, among others: grain working time and the state of wear of active grain vertexes, the intensity of fracturing and the number of active edges. The working time of the grain is the period of time from the moment when the grain began to work periodically in contact with the workpiece. In the developed models, it was assumed that at the moment of fracture, the grain is replaced with a grain that has not been working so far, with the same properties in a statistical sense.

The expected number of fractures could be represented by the equation:

(18) $$\sum_{j=1}^{t-1}u_{t=j}\left(1-p_{k=t-j}\right)$$

where: by $u_{j}$ is the expected number of fractures at time *j*. $p_{t}-j$ is the probability that the grain starting work at time *j* will not be fractured in j…t, i.e., k=t−j. From Formula (21) it is possible to determine the number of grains in groups with a fixed working time *t* at time k=t−j, for j=1,2,⋯,t−1:

(19) $$n_{k=t-j}\left(t\right)=u_{t=j}p_{k=t-j},\;dla\;j=1,2,\cdots ,t-1.$$

The characteristic group for determining the durability of the grinding wheel are grains working from the beginning. Their working time will be $t_{z}=t_{a}+n$; n=0,1,2,3,⋯, determined for grains that will work *n* time units.

The number of grains over time t+n>t, in groups with a fixed duration of *k* greater than *t*, depends on the distribution of the grains that worked *t* time units. If by $p_{t}$ we denote the probability that the working time of the grain exceeded *t* units, then the conditional probability that the grain will not fractured after the working time t+n time units is $p_{t}+\frac{n}{p_{t}}$.

The number of grains working from the beginning (grains whose working time is t+n time units, for n=0,1,2,3,⋯) is equal to:

(20) $$n_{k=t+n}=n_{k=t}=\frac{p_{k=t+n}}{p_{k=t}},\;dla\;n=0,1,2,\cdots p_{k=t+n}<p_{k=t}$$

**Figure 1:**
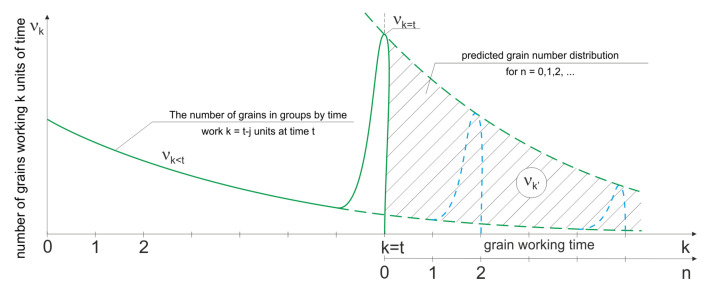
The determined (up to k=t) and predicted (k>t) distribution of the number of grains at the n=0,1,2,….

Figure 1 shows an exemplary plot of the known (up to the time k=t) and the predicted distribution of the number of grains in successive moments.

The expected number of fractures at time *t*, in the group of grains working from the beginning, is equal to:

(21) $$b_{t}=n_{k=t}\left(1-p_{k=t}\right)$$

where $n_{k}$ is the number of grains with a working time of k=t units, $p_{k=t}$ is the probability that the working time of the grain will exceed *t* units.

The total expected number of fractures at time *t* is the sum of the fractures from the group of grains working from the beginning and the group of grains that started work in the following moments of the grinding process. In addition, the total number of fractures is the number of grains with a working time of zero units:

(22) $$u_{t}=n_{k=0}\left(t\right)=\sum_{j=1}^{t-1}u_{t=j}\left(1-p_{k=t-j}\right)+n_{k=t}\left(i=p_{k=t}\right)$$

The expected number of fracturing in *n* consecutive moments is:

(23) $$u_{t=t+n}=\sum_{j-1}^{t-1}u_{t=j}\left(1-p_{k=t-j}\right)+\sum_{i=0}^{n}n_{k=t}\left(1-\frac{p_{k=t+n}}{p_{k=t}}\right)$$

When determining the distribution of the number of grains in groups with a specific working time k≥t, it was assumed that at the start of the grinding process the working time of all grains on the active surface of the grinding wheel is zero and their number is defined by $n_{0}\left(t_{0}\right)=n_{k=0}\left(t=0\right)$. The total duration of the process is *t* units. Then the distribution of the working time of grains and their number in groups with a fixed working time is defined as follows:

(24) $$n_{k}\left(t\right)=\left\{\begin{array}{c} u(t),k=0\\ u_{\left(t-k\right)}\cdot p_{k},k=1,2,\ldots ,t-k\\ v_{0}\left(t_{0}\right)-\int_{i=1}^{t-1}v_{i}\left(t\right),k=t\\ 0,t>0 \end{array}\right.$$

(25) $$u_{t}=v_{k=0}\left(t\right)=\int_{i=1}^{t}\left(1-p_{k=i}\right)$$

**Figure 2:**
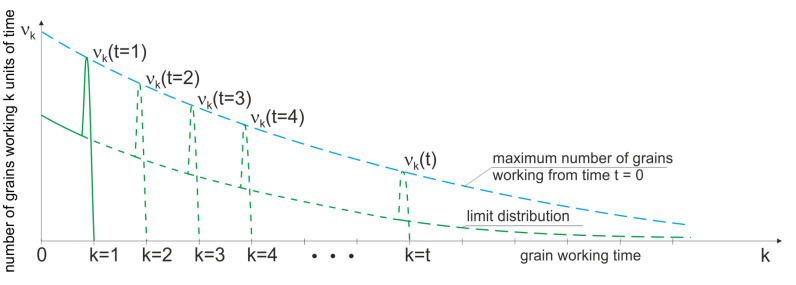
The distribution of the number of grains in successive moments *t* of the grinding process.

Grain fracturing, occurring not necessarily with a constant intensity, causes that the time distribution of active grains is variable during the grinding process (Figure 2). If the probability of grain fracturing does not depend on the grain operation time *k*, then quite fast stabilization of the operating time distribution $n_{k}\left(t\right)$ takes place. The distribution of active grains working time quickly approaches the limit distribution.

### 2.5. Tool Wear in Conditions of Intensive Fracturing of Abrasive Grains

Under operating conditions of grinding wheels characterized by a significant intensity of self-sharpening, which is the cause of  tool wear, the fracturing of grains from the surface of the grinding wheel could be treated as a stationary process and without any sequelae. It was assumed that the distribution of the number of fractures in the time from $t_{0}$ to $t_{1}$ does not depend on the distribution of the number of fractures until $t_{0}$, because the time of a single grain load is small, and the number of simultaneously loaded grains is very small compared to the total number of active grains. The time the grain is in contact with the workpiece $k_{0}$ is very short in relation to the time of one revolution of the grinding wheel. Analyzing the grain fracturing at time *t*, it could be assumed that it would not affect that part of the active surface of the grinding wheel that will be in contact with the workpiece at time t+Δt if the condition $\Delta t>k_{0}$ is met. The occurrence of successive fractures could be treated as independent phenomena, and the fracturing stream as a Poisson stream. The probability that in the period $t_{s}=t_{1}-t_{0}$ there will be n=1,2,… fractures is as follow:

(26) $$P\left\{N\left(t_{s}\right)=n\right\}=\frac{{\left(\lambda t_{s}\right)}^{n}}{n!}exp\left(\lambda t_{s}\right)$$

**Figure 3:**
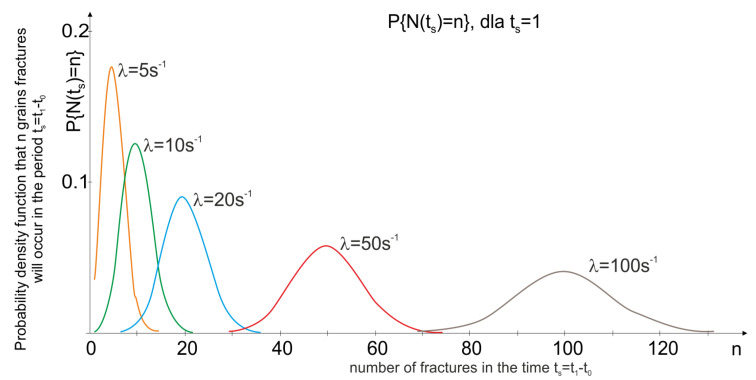
Probability density function that *n* grain fractures will occur in the period $t_{s}=t_{1}-t_{0}$.

The graph of dependence $P\left\{N\left(t_{s}\right)=n\right\}=f\left(n\right)$ for the time period $t_{s}=1$, for different values of λ of the expected number of fractures, is shown in Figure 3.

### 2.6. Total Attritious Wear Area and Volumetric Wear

Knowing the time distribution of active grains, it is possible to determine the total area of the grain attritious wear and volumetric wear of the grinding wheel. The attritious wear area of a given active grain depends on its load, wear resistance and working time. It could be assumed that the attritous wear area of the grain $A_{k}\left(t\right)$ with a working time of *k* units at time *t* is:

(27) $$A_{k}\left(t\right)=C_{z}a_{z}\left(k\right)$$

where $C_{z}$ denotes the constant, and $f_{z}\left(k\right)$ is the function describing the dependence of the grain attritious wear area as a function of its work time *k*.

After taking into account the distribution of the grain working time, the total attritious wear area of the grinding wheel can be determined:

(28) $$A_{s}\left(t\right)=\sum_{k}A_{k}\left(t\right)n_{k}\left(t\right)=C_{z}\sum_{k}a_{z}\left(k\right)n_{k}\left(t\right)$$

In the case of low fracture intensity, the working time of the grains is long. The working time distribution is changing because of increasing number of grains with a long activity time and the total area of the attritious wear is increased. $A_{s}\left(t\right)$ is only slightly smaller than the value of the expression $A_{s}\left(t\right)=C_{z}a_{z}\left(k\right)n_{k=t}\left(t\right)$.

In the case of intensive fracture wear of grains, when the distribution of the grain working time after a short period of grinding is close to the stationary distribution, the total attritious wear area after some time reaches the values corresponding to the limit distribution:

(29) $$\lim_{t\to \infty }A_{s}\left(t\right)=C_{z}N_{a}\mu_{w}^{-1}\sum_{k}p_{k}a_{z}\left(k\right)$$

where $\mu_{w}$ is the average time for grain fracturing, and $N_{a}$ is the number of active grains, taken as the constant value during grinding.

The attritious wear of the grains is the reason for a slight reduction in the volume of the grinding wheel. The main reason for the changes in the volume of the grinding wheel is grain fracturing, which can be described by a relationship that includes the total number of pull-outs from groups of grains with a specified working time:

(30) $$u_{n}\left(t\right)=\sum_{t}\left[\sum_{j=1}^{t-1}u_{t=j}\left(1-p_{k=t-j}\right)+n_{k=t}\left(1-p_{k=t}\right)\right]=\sum_{t}u_{t}.$$

The ${\bar{V}}_{z}$ determines the average volume of the pulled-out grains, and $V_{z}^{\left(1\right)}$ determines the grain size. The reduction in the volume of the grinding wheel as a result of grain fractures and binder breaks is following:

(31) $$\Delta V=\frac{{\bar{V}}_{z}}{V_{z}^{\left(1\right)}}u_{n}\left(t\right)=\frac{{\bar{V}}_{z}}{V_{z}^{\left(1\right)}}\sum_{t}\sum_{k}n_{k}\left(1-p_{k}\right)$$

The above analysis is limited to the determination of general relationships, without determining the exact influence of the grain working time on the probability of its fracture.

## 3. Materials and Methods

### 3.1. Experimental Research

**Figure 4:**
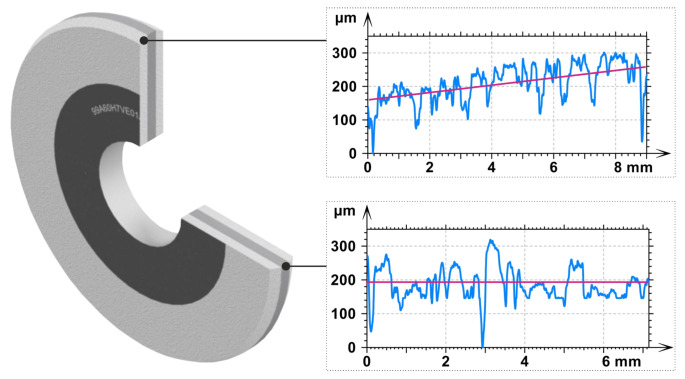
The shape of the layered grinding wheel used in the experimental tests.

During the research, the grinding process of surfaces with intermittent feed was carried out. For this purpose, a numerically controlled grinding machine FAS SGP 250 CNC was used. The samples were ground using a tool with chamfered external edges on both sides (Figure 4). That allowed for highly efficient processing of workpieces. Despite the large contact depth of $a_{e}=0.1$ mm, the value in individual passes was smaller as a result of the tools profile. The parameters of the experimental studies are summarized in Table 1. The assumed grinding parameters (resulting in high loading of abrasive grains) allow for the intensification of the grinding wheel wear.

The applied machining strategy allowed for the material removal and sparking passes. The workpiece was processed with a grinding speed of 20 m/s and a transverse feed of 1 mm. The shape of the tool was measured using an optical scanner after every five removed layers of the material (each after t=608 s of grinding). The first measurement was performed after dressing (N=0 grinding passes, t=0 s). The last one was performed after N=15 removed layers (t=1824 s).

**Figure 5:**
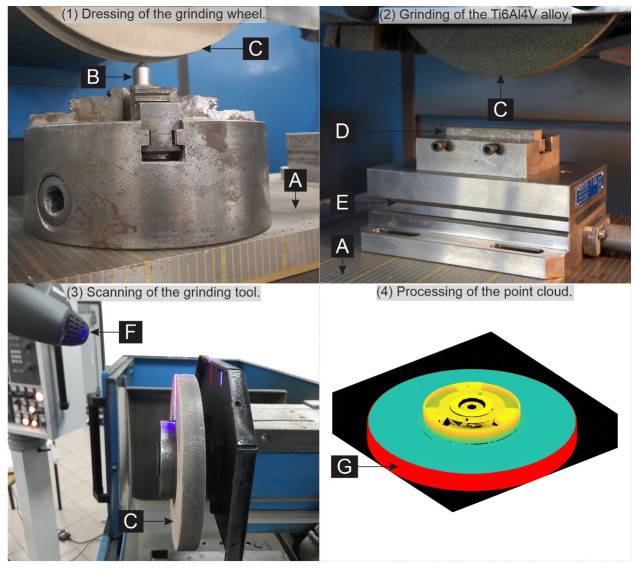
Methodology of the experimental research; **A**—magnetic table, **B**—single grain dresser, **C**—grinding wheel, **D**—workpiece, **E**—sample holder, **F**—scanning head, **G**—scanned point cloud.

After each N=5 (t=608 s) the grinding wheel active surface was scanned. The tool was not disassembled from the spindle during the scanning. That allowed for comparable results because the tool was not required to be balanced and dressed each time. The processed sample was also one and not disassembled during the entire cycle. The applied measurement method is shown in Figure 5.

The conical chamfer was made with a 3-carat, single-grain diamond dresser, mounted in a four-jaw chuck directly under the tool, as presented in Figure 5. The measured point clouds were imported to the MathWorks Matlab environment, where the grinding wheel radial and volumetric wear was calculated.

The workpiece material was titanium alloy Ti6Al4V, which is considered to be difficult to machine. That material is widely used for the production of an aircraft engine components. The applicability is determined by high mechanical strength at high operating temperatures. During the grinding process that could also intensify the wear of the abrasive tools. The samples with dimensions of 80 mm (L) × 13 mm (W) × 20 mm (H) were prepared.

### 3.2. Simulation Research

Simulation tests of the grinding wheel wear process were carried out using the assumptions described in Section 2 implemented in Matlab. The Algorithm 1 (in pseudo-code) is presented below.
**Algorithm 1:** Algorithm to analysis of abrasive grains wear processes
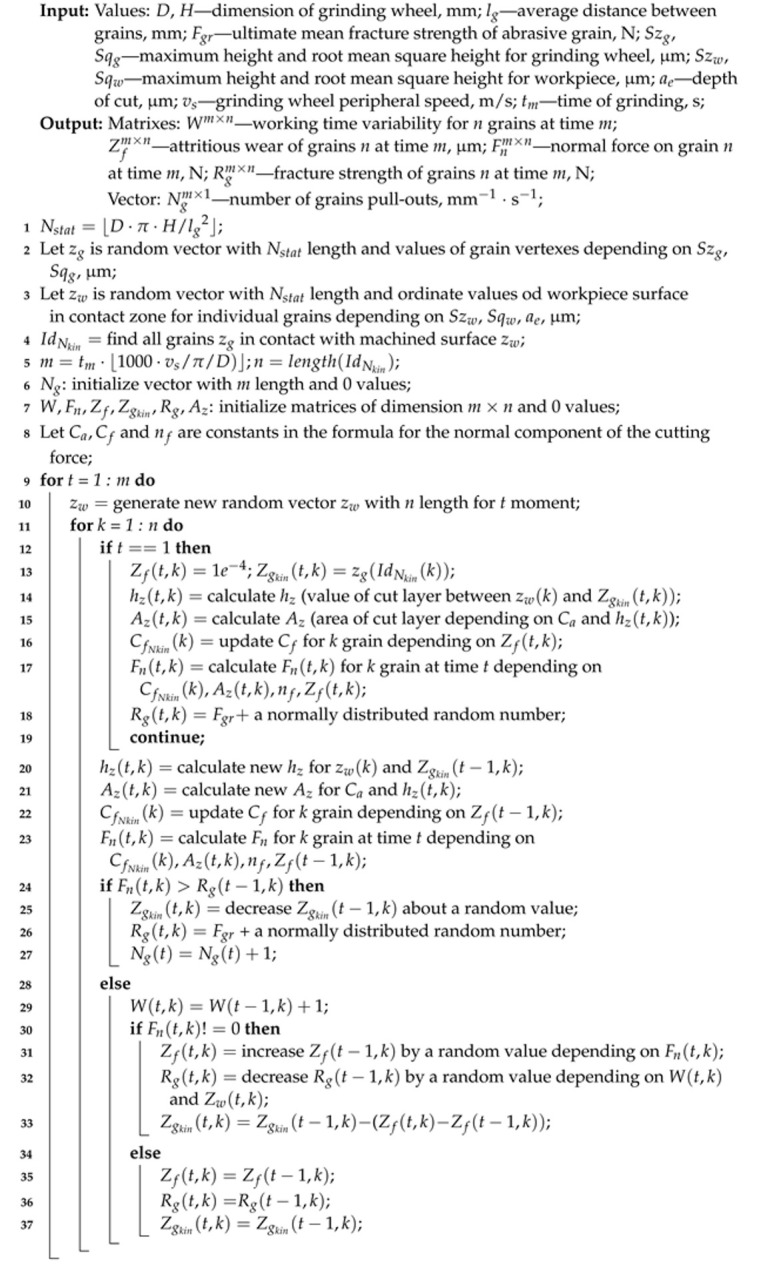


## 4. Results and Discussion

**Figure 6:**
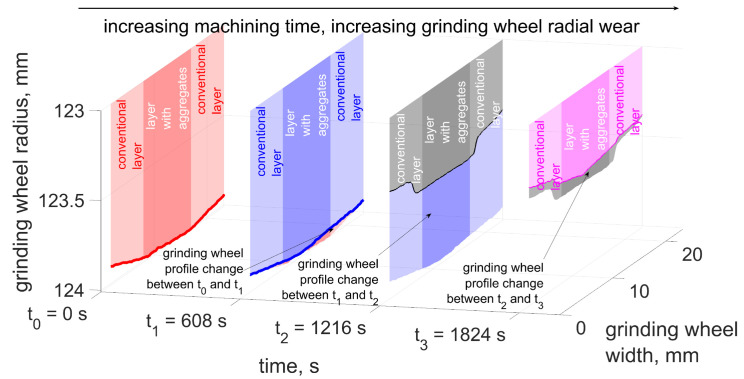
Change in radial wear of the grinding wheel during grinding.

The machining process was carried out with a large depth of cut ($a_{e}$ = 0.1 μm). The tapered chamfer of the grinding wheel (conventional layer) distributed the size of the allowance into successive zones, reducing the depth of cut in each grinding zone to approximately 10 μm. The inner layer (with the addition of abrasive aggregates) made the workpiece surface smooth. The grinding wheel profile was measured at the beginning of the process (*t* = 0 s) and in three subsequent stages (each after removing the next five layers of material—5 · 0.1 μm), for *t* = 608 s, *t* = 1216 s and *t* = 1824 s. The results of the grinding wheel profile measurements are shown in Figure 6.

**Figure 7:**
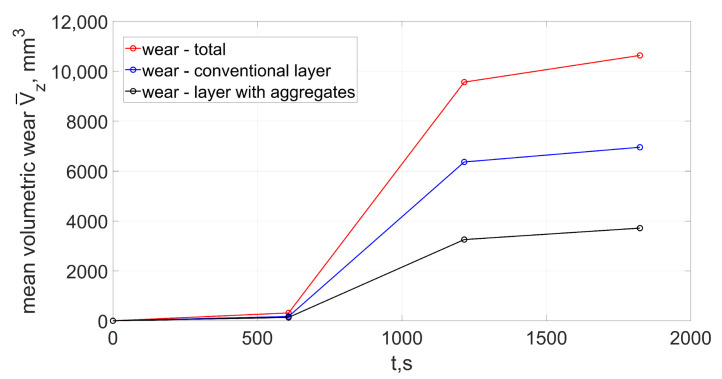
Total mean volumetric wear ${\bar{V}}_{z}$ of the grinding wheel (Equation (31)).

The selected machining parameters resulted in grinding with large cross-sections of the removed layers in order to initiate the process of abrasive grains fracturing. During the first 600 s of machining small radial wear was observed mainly in the most protruded part of the wheel, which was active during the process. The maximum value of radial wear was $\Delta r_{max}$ = 43 μm, and the mean value $\Delta r_{mean}$ = 16 μm. In the following 600-s period rapid radial wear occurred ($\Delta r_{mean}$ = 480 μm). The wear progressed from the outermost layers of the wheel. Fractures of grains in these zones meant that they were able to remove less than the required volume of the allowance. That resulted in an increase of the cross-sectional area of the removed layers by abrasive grains in subsequent areas of the grinding wheel. In a short time the macro fracturing of the abrasive grains occurred or their complete pull-out from the bond. As a result, there was a rapid volumetric wear of the wheel (Figure 7). In the last stage, the progressive radial wear caused the reformation of the conical chamfer on the active surface of the grinding wheel. Radial wear decreased ($\Delta r_{mean}$ = 55 μm). The outer zones of the grinding wheel were again most heavily loaded.

In the first stage (from 0 s to 608 s) a slight volumetric wear was observed (${\bar{V}}_{z}$ = 316 mm^3^). During this period, the value of the grinding ratio *G* (calculated as the ratio of the volume of material removed to the volumetric wear of the grinding wheel) was 1.65. In the next stage, as a result of the rapid process of grain fracture, a large increase in volumetric wear occured and the value of the grinding ratio droped to values unacceptable for grinding processes.

**Figure 8:**
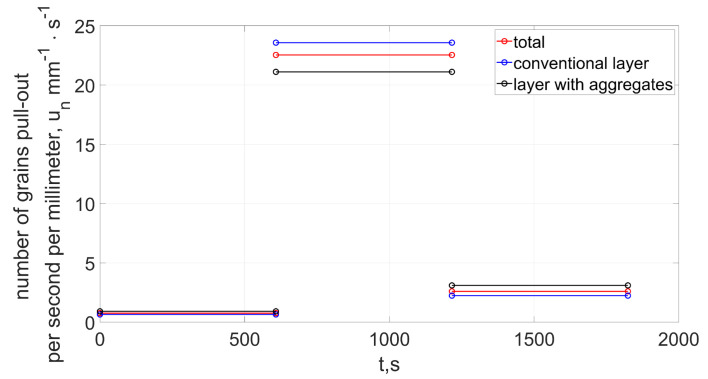
Number of fractured (pulled-out) abrasive grains (Equation (30)).

The number of grain fractures in the first stage was of low intensity (Figure 8). The average number of fractures in the time from 0 to 608 s was 0.77 mm^−1^ · s^−1^. This intensity (taking into account the average size of the abrasive grain) was similar for both the conventional layer and the layer with the addition of abrasive aggregates.

An increase in the intensity of grain fractures occurred in the period from 608 to 1216 s. During this period, the number of fractures increases almost 30 times, leading to an intensive increase in the volumetric wear of the grinding wheel. The outer zones of the grinding wheel were crushed, which were responsible for the removal of subsequent layers of the allowance along with the subsequent transverse feed of the tool. Fracturing was caused by an increase in the load on external grains (as a result of an increase in the cross-sectional area of the cut layers) exceeding the strength of the abrasive grains or the bond [14,17]. The zone of intense fracturing moved from the outer zones of the tool toward its center (Figure 6). As a result, the profile of the grinding wheel was similar to the original shape, and at the same time the number of fractures decreased to a value of about mm^−1^ · s^−1^.

**Figure 9:**
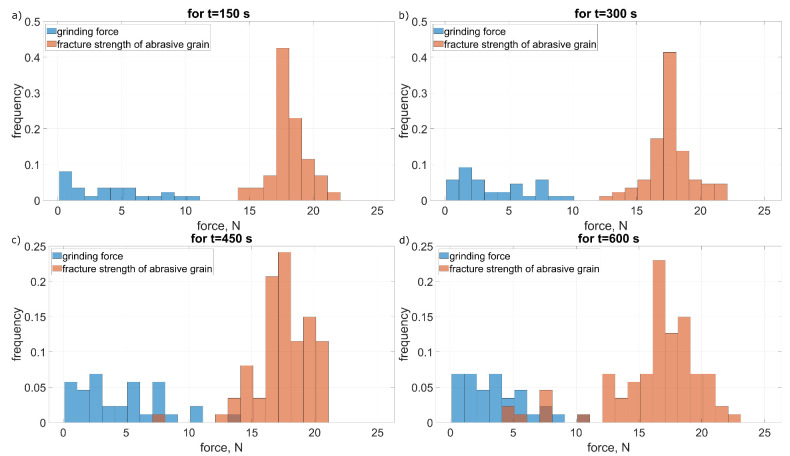
Grinding forces distribution $F_{z}$ (Equation (15)) and fracture strength $w_{z}$ (Equation (16)) of the abrasive grain for low grain fracture (pull-out) intensivity at: (**a**) 150 s, (**b**) 300 s, (**c**) 450 s and (**d**) 600 s of grinding time.

**Figure 10:**
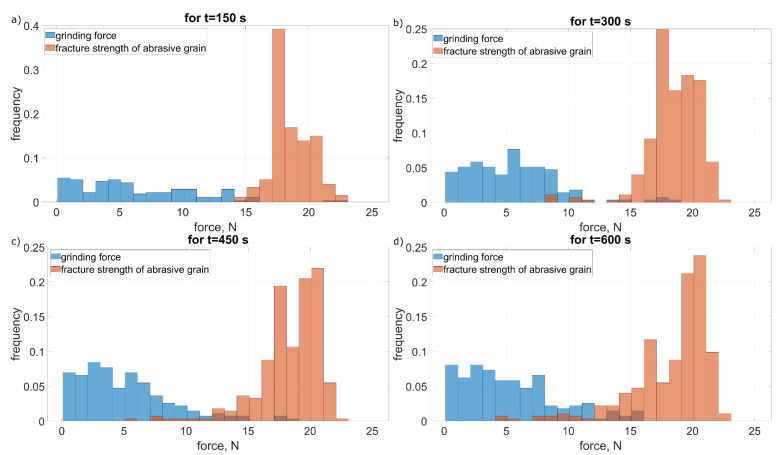
Grinding forces distribution $F_{z}$ (Equation (15)) and fracture strength $w_{z}$ (Equation (16)) of the abrasive grain for high grain fracture (pull-out) intensivity at: (**a**) 150 s, (**b**) 300 s, (**c**) 450 s and (**d**) 600 s of grinding time.

In Figure 9 and Figure 10 changes in the distribution of the values of grinding forces and strength of abrasive grains for the intensity of fracturing amounting to 0.77 mm^−1^ · s^−1^ (Figure 9) and 22 mm^−1^ · s^−1^ (Figure 10) are shown. In both cases, there was an increase in the frequency of fractures along with the time of machining. This increase was the result of a reduction in grain strength as a result of increasing mechanical and thermal loads after their multiple contacts with the workpiece [23]. The change in the intensity of fractures, with the same parameters of the grain strength distribution, was the result of an increase in the cross-sections cut by individual abrasive grains. As a result, there was a large increase in the value of cutting forces and more often the limit value of the strength of the abrasive grains was exceeded (Figure 10). As a result, the intensity of fracturing increased [10].

The increase in the intensity of fractures caused that the zone of the grinding wheel in contact with the workpiece did not remove the entire allowance. As a result, the successive zones of the grinding wheel in contact with the workpiece were subject to higher loads. This led to a movement of the zone of intense fractures from the outer, most loaded zones to its center (Figure 6). A ‘step’ was formed on the active surface of the grinding wheel, the width of which decreased with the time of grinding. The moving front of intense fracturing significantly increased the volumetric wear of the grinding wheel. The wear intensity decreased when the zone of intense fractures moved to the center of the grinding wheel. The resulting conical chamfer ensured a more even distribution of the allowance for individual zones of the grinding wheel [30]. This resulted in a reduction in the intensity of fractures. However, as the outer longest grinding zones of the tool began to fracture more intensively, the volumetric wear process increased again.

**Figure 11:**
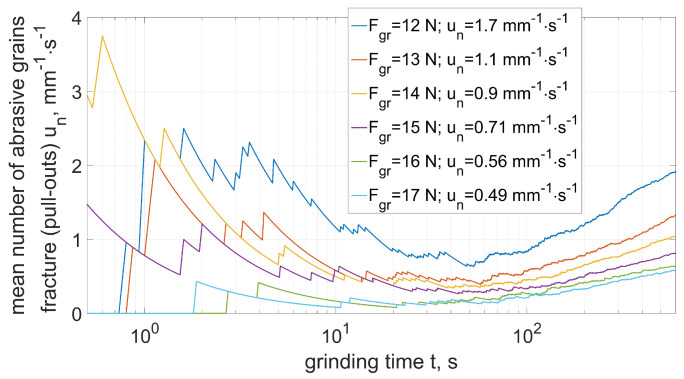
Mean number of abrasive grains fracture (pull-out) depending on grain strength (Equation (30))—case of low intensity (period from $t_{0}$ to $t_{1}$).

**Figure 12:**
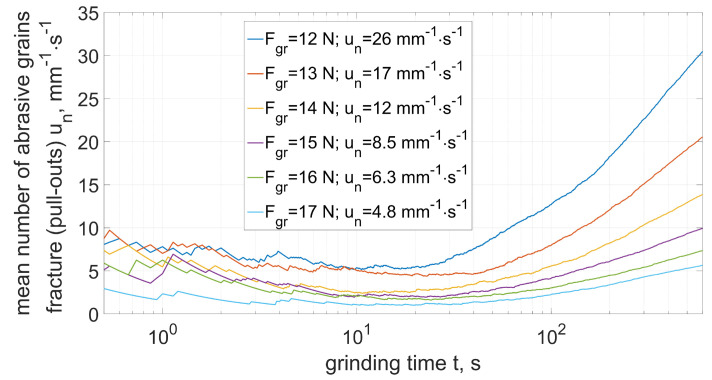
Mean number of abrasive grains fracture (pull-out) depending on grain strength (Equation (30))—case of high intensity (period from $t_{1}$ to $t_{2}$).

The results of the analysis of the intensity of fracturing depending on the strength of the abrasive grains are shown in the Figure 11 and Figure 12.

In the case of lower intensity of the fractures (Figure 11) they dominate in the first contact stage (especially in the case of grains with lower strength). Along with the increase in the machining time, a slight increase in the number of fractures was observed due to the decreasing strength of the grains repeatedly entering the grinding zone. In the case of high intensity of fracturing (Figure 12), an increase in the number of chipping was observed along with the grinding time. This increase was a product of the increased mechanical and thermal loads of the abrasive grains (Figure 10).

The probabilistic features of the grinding process are largely due to the random phenomena of attrition and fractures of the abrasive grains and the pull-out of the whole grains. The physical properties and geometrical features of the individual grains vary considerably. Abrasive wear of the grains is an additive process, dependent on the increasing number of contacts and the impact forces in individual contacts. The fracture strength of the grain decreases with the accumulation of the effects of successive loads on individual grains. The experimental determination of the state of active grains on the grinding wheel surface is limited due to the large number of grains and the possibility of identification without moving the tool to the measuring station.

**Figure 13:**
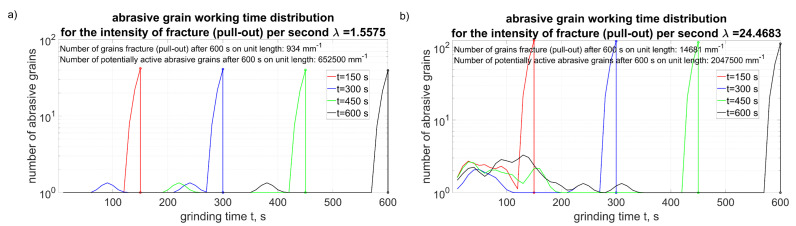
Abrasive grain working time distributions (Equation (24)) for variable intensity of fracturing: (**a**) low intensity λ=1.56, (**b**) high intensity λ=24.47.

Figure 13 shows the distribution of the working time of the grains for different intensities of the fractures. The number of grains with a specified working time on the ordinate axis was included in a logarithmic scale to show a relatively small number of “new” grains, i.e., those that became active after fractured neighboring ones.

Modeling the distribution of grain grain working time and the analysis of attrition and fracturing processes took into account the events involving the mechanical load exceeding the strength limit, depending on the height of the apex of the grain, as well as the effects of accumulation of loads and the effect of the abrasion of the grain tip on the cutting force.

For the simulation of the load and modeling the levels of immediate strength and resistance to the sum load of grains, random functions with a given convolution of distributions characteristic for the analyzed tools were used. The obtained results for specific relations regarding load and strength, working time and the location of grains on the active surface of the tool allowed for a detailed analysis of the grinding wheel wear process based on:

- determined distribution of grain working time,
- the number of grains in groups with a specified working time,
- the number of fracturing in groups of grains with a specified working time,
- changes in the intensity of fractures during the wheel life cycle.

The developed methods of modeling the wear processes made it possible to extend the time scale, to learn about intermediate states, and to distinguish factors whose influence was hidden in the complex process of interactions accumulation. An important advantage was the possibility of estimating the dispersion of the values of non-measurable features, while experimentally only average values could be determined. The simulation of the abrasive tool wear process made it possible to analyze it for various tool operating conditions. In order to present the possibility of conducting simulation tests, calculations were carried out for various conditions with different intensity of abrasive grain fractures during the grinding process.

The developed models and simulation procedures took into account that the probabilistic nature of the features and properties of the abrasive tools was the result of: randomness of the size, shape, microgeometry, apex radii and apex angles of the grains, random orientation and location of the grains on the active surface of the grinding wheel, macro inhomogeneity of the grinding wheel, randomness of the grinding wheel grain strength and bonding forces between the grains and the binder, as well as the randomness of the grain load.

In experimental tests, the processed material was titanium alloy Ti6Al4V. It was shown that the change in the shape of the grinding zone as a result of volumetric wear, subject to its expansion and gradual movement toward the central zone, causes a significant change in the intensity of fractures. In the case of the multilayer tools tested, it was also dependent on the properties of individual layers and their variable participation in the process of shaping the machined surface.

The attritious wear of grains is an additive process, dependent on the increasing number of contacts and the forces of interaction in individual contacts. The fracture strength of the grains decreased with the accumulation of the effects of successive loads on individual grains. The experimental determination of the state of active grains on the grinding wheel surface was limited due to the large number of grains and the possibility of identification without moving the tool to the measuring station.

Modeling the distribution of abrasive grain working time and the analysis of attrition and fracturing processes took into account the events involving the mechanical load exceeding the strength limit, depending on the height of the apex of the grain, as well as the effects of accumulation of loads and the effect of attrition of the grain tip on the cutting force.

The obtained modeling results for specific relations regarding the load and strength, working time, and the location of the grains on the active surface of the tool allowed for a detailed analysis of the grinding wheel wear process based on the temporary values of fractures in groups of grains with a specific working time and statistical parameters of abrasive grain wear characteristics over machining time and changes in the distribution of the range of working of the tips of active grains on the grinding wheel surface.

The application usefulness of the presented methodology of analyses lies in the fact that modeling with the use of even a small set of empirical data allows to determine the values of other quantities, the experimental determination of which is very difficult and time-consuming. By measuring the volumetric wear of the grinding wheel after a specific grinding time, it is possible to calculate the average number of crushed grains, then the intensity of fractures, and then the distribution of active grains working time and the total grinding surface of the grinding wheel. Therefore, you can determine those features that allow you to better understand and describe temporary states and their changes along with the increase in the machining time. This allows to reduce the cost of developing new tools dedicated to specific applications.

## 5. Conclusions

In this research, the probabilistic aspects of modeling and analysis of grinding wheel wear as well as the results of experimental tests of peripheral grinding with grinding wheels with a different zone structure are presented. Based on the presented results of experimental and analytical research the following conclusions were drawn:

- In the analyzed grinding process of the Ti6Al4V titanium alloy, two states of grinding wheel volume wear with different intensity were observed: (i) the state of low intensity of fracturing (number of fractures $u_{n}$ about 1 mm^−1^ s^−1^), (ii) the state of rapid volumetric wear (number of fractures $u_{n}>$ 20 mm^−1^ s^−1^).
- The change in the intensity of fracturing is the result of a change in the load on the abrasive grains, especially in the middle zone of the three-zone grinding wheel. The outer zones have a conical shape. The shape wear zone that moves from the outer zones to the center of the grinding wheel causes the allowance to be removed when the central zone is increased. This causes an increase in the load on the grains in this zone for a relatively short time, during which the radius of the central zone will not decrease by the amount resulting from the wear of the outer zones. After this time, the process stabilizes.
- More than a twofold increase in the load on abrasive grains (as a result of an increase in the allowance, and thus mechanical and thermal loads) causes a temporary increase in the intensity of abrasive grain fracturing, even 10 times to 20 times. This is due to the different characteristics of the center zone of the grinding wheel, in which abrasive aggregates are present, ensuring the reduction of the specific grinding energy in this zone. The central zone is the zone intended for smoothing the machined surface. After a short time, the radius of the central zone aligns with the value of the chamfer radius in the adjacent zone and the intensity of fracturing stabilizes at a lower level.
- The inclusion of abrasive grain fracturing in the modeling process of the grinding wheel wear as a result of exceeding the permissible load of the abrasive grains and changes in their load over time enables a detailed analysis of the distribution of the working time of the abrasive grains. The selection of modeling parameters for the configuration of the tool and the workpiece is preceded by an experimental determination of the volumetric wear of the grinding wheel in individual time intervals.

The developed model of the grinding wheel wear process was used to analyze the grinding process with the use of the zone-diversified grinding wheel with different shape of the active surface in individual zones. This analysis makes it possible to identify the causes of a short-term increase in the intensity of abrasive grain fracturing, and then stabilization at a low level. Tools with a zone-diversified structure allow improving the surface quality without reducing machining efficiency and additionally allow reducing the process specific energy (which is the subject of further work). An important role is also played by the shaping of the outer zones in the form of a cone. It can be concluded that the use of a conical chamfer with a width smaller than the width of the outer zones has a positive effect on stabilizing the intensity of fracturing from the beginning of the wheel life and the grinding wheel working life is extended.

## Figures and Tables

**Table 1:** Conditions and parameters of grinding tests.

Machining station	
Grinding machine	FAS SGP 250 CNC
Grinding wheel dimensions	250 × 25 mm
Chamfer of the grinding wheel edges	on both edges 100 µm × 9 mm
Grinding wheel characteristics	99A60K7V
**Grinding process parameters**	
Grinding speed	20 m/s
Depth of cut	0.1 mm
Transverse feed	1 mm
Workpiece speed	3 m/s
Cooling method	Flood cooling EMU 12 with 5% solution
**Optical scanning equipment**	
Scanning tool	3D scanner Nikon ModelMaker h120
The measurement frequency	450 Hz
Resolution	35 µm

## Data Availability

Not applicable.

## References

[1] Guo G., Malkin S. (2008). Grinding Technology Theory and Applications of Machining with Abrasives.

[2] Ezugwu E.O., Bonney J., Yamane Y. (2003). An overview of the machinability of aeroengine alloys. J. Mater. Process. Technol..

[3] Ezugwu E.O., Bonney J., Fadare D.A., Sales W.F. (2005). Machining of nickel-base, Inconel 718, alloy with ceramic tools under finishing conditions with various coolant supply pressures. J. Mater. Process. Technol..

[4] Tomlinson W.J., Blunt L.A., Spraggett S. (1991). The effect of workpiece speed and grinding-wheel condition on the thickness of white layers formed on EN. 24 ground surfaces. J. Mater. Process. Technol..

[5] Malkin S., Cook N.H. (1971). The Wear of Grinding Wheels: Part 1—Attritious Wear. J. Eng. Ind..

[6] Malkin S., Cook N.H. (1971). The Wear of Grinding Wheels: Part 2—Fracture Wear. J. Eng. Ind..

[7] Godino L., Pombo I., Sanchez J.A., Alvarez J. (2018). On the development and evolution of wear flats in microcrystalline sintered alumina grinding wheels. J. Manuf. Process..

[8] Nadolny K. (2015). Wear phenomena of grinding wheels with sol–gel alumina abrasive grains and glass–ceramic vitrified bond during internal cylindrical traverse grinding of 100Cr6 steel. Int. J. Adv. Manuf. Technol..

[9] Inasaki I., Karpuschewski B., Lee H.-S. (2001). Grinding Chatter—Origin and Suppression. CIRP Ann..

[10] Badger J., Murphy S., O’Donnell G. (2011). The effect of wheel eccentricity and run-out on grinding forces, waviness, wheel wear and chatter. Int. J. Mach. Tools Manuf..

[11] Kacalak W., Lipiński D., Szafraniec F., Zawada-Tomkiewicz A., Tandecka K., Królczyk G. (2020). Metrological basis for assessing the state of the active surface of abrasive tools based on parameters characterizing their machining potential. Measurement.

[12] Lipiński D., Banaszek K., Rypina Ł. (2022). Analysis of the Cutting Abilities of the Multilayer Grinding Wheels—Case of Ti-6Al-4V Alloy Grinding. Materials.

[13] Klocke F., Barth S., Wrobel C., Weiß M., Mattfeld P., Brakhage K.H., Rom M. (2016). Modelling of the grinding wheel structure depending on the volumetric composition. Procedia CIRP.

[14] Stępień P. (2009). A probabilistic model of the grinding process. Appl. Math. Model..

[15] Tianyu Y., Ashraf F., Bastawros A.C. (2017). Experimental and modeling characterization of wear and life expectancy of electroplated CBN grinding wheels. Int. J. Mach. Tools Manuf..

[16] Nosenko V.A., Fedotov E.V., Nosenko S.V., Danilenko M.V. (2009). Probabilities of Abrasive Tool Grain Wearing during Grinding. J. Mach. Manuf. Reliab..

[17] Novoselov Y., Bratan S., Bogutsky V. (2016). Analysis of Relation between Grinding Wheel Wear and Abrasive Grains Wear. Procedia Eng..

[18] Tonshoff H.K., Peters J., Inasaki I., Paul T. (1992). Modelling and Simulation of Grinding Process. CIRP Ann..

[19] Marinescu I.D., Rowe W.B., Dimitrov B. (2004). Tribology of Abrasive Machining Processes.

[20] Brinksmeier E., Aurich J.C., Govekar E., Heinzel C., Hoffmeister H.W., Klocke F., Peters J., Rentsch R., Stephenson D.J., Uhlmann E. (2006). Advances in Modeling and Simulation of Grinding Processes. CIRP Ann..

[21] Lipiński D., Kacalak W., Bałasz B. (2019). Optimization of sequential grinding process in a fuzzy environment using genetic algorithms. J. Braz. Soc. Mech. Sci. Eng..

[22] Rypina Ł., Lipiński D., Bałasz B., Kacalak W., Szatkiewicz T. (2020). Analysis and Modeling of the Micro-Cutting Process of Ti-6Al-4V Titanium Alloy with Single Abrasive Grain. Materials.

[23] Bergs T., Ohlert M., Prinz S., Barth S. (2020). Modelling of Fracture Behavir of CBN Grains during Single Grain Dressing using FEM. Procedia CIRP.

[24] Li H.N., Axinte D. (2017). On a stochastically grain-discretised model for 2D/3D temperature mapping prediction in grinding. Int. J. Mach. Tools Manuf..

[25] Li H.N., Yu T.B., Wang Z.X., Zhu L.D., Wang W.S. (2017). Detailed modeling of cutting forces in grinding process considering variable stages of grain-workpiece micro interactions. Int. J. Mech. Sci..

[26] Aurich J.C., Kirsch B. (2012). Kinematic simulation of high-performance grinding for analysis of chip parameters of single grains. CIRP J. Manuf. Sci. Technol..

[27] Zhao B., Xiao G., Ding W., Li X., Huan H., Wang Y. (2020). Effect of grain contents of a single-aggregated cubic boron nitride grain on material removal mechanism during Ti–6Al–4V alloy grinding. Ceram. Int..

[28] Wu J., Cheng J., Liu B. (2022). Research on the grinding performance of a defined grain arrangement diamond grinding wheel with small grit (95 μm). Int. J. Adv. Manuf. Technol..

[29] Brecker J.N. (1974). The Fracture Strength of Abrasive Grains. J. Eng. Ind..

[30] Nadolny K., Słowiński B. (2011). The Effects of Wear upon the Axial Profile of a Grinding Wheel in the Construction of Innovative Grinding Wheels for Internal Cylindrical Grinding. Adv. Tribol..

